# Cannabidiol (CBD): A Systematic Review of Clinical and Preclinical Evidence in the Treatment of Pain

**DOI:** 10.3390/ph17111438

**Published:** 2024-10-28

**Authors:** Guillermo Cásedas, Martín de Yarza-Sancho, Víctor López

**Affiliations:** 1 Department of Pharmacy, Faculty of Health Sciences, Universidad San Jorge, 50830 Zaragoza, Spain; gcasedas@usj.es (G.C.); martinyarza98@gmail.com (M.d.Y.-S.); 2 Instituto Agroalimentario de Aragón-IA2, CITA-Universidad de Zaragoza, 50009 Zaragoza, Spain

**Keywords:** cannabis, pain, CBD, analgesic, clinical trials, cannabinoids

## Abstract

**Background/Objectives**: *Cannabis sativa* L. is a plant that has been used for thousands of years for its industrial and medicinal properties. In recent years, there has been a rise in the study of this plant due to its bioactive compounds for pharmaceutical applications. Particularly, cannabidiol has demonstrated analgesic and non-psychoactive properties. The objective of this systematic review is to update and to gather the clinical and preclinical evidence on CBD in pain treatment. **Methods**: This study was performed following the PRISMA guidelines and using the following search terms “((cannabidiol) NOT (THC)) NOT (tetrahydrocannabinol)) AND (pain treatment)” in PubMed and Web of Science, with the following inclusion criteria: CBD pain treatment without THC in monotherapy, including both clinical and preclinical trials. From the initial sample of more than 500 articles, a total of 40 studies were selected, eliminating duplicate studies from the databases and considering the inclusion and exclusion criteria. On one hand, clinical trials were analyzed using CBD products without THC used in monotherapy, assigning a Jadad score to evaluate the quality/bias of the trials; on the other hand, the main preclinical trials were analyzed, grouping the results into in vivo and in vitro trials. **Results**: Based on the review conducted, there is sufficient clinical and preclinical evidence of CBD in pain treatment, so CBD could be an effective and safe treatment in reducing pain due to its analgesic and anti-inflammatory properties. These effects appear to be primarily mediated by the activation of TRPV-1, 5HT-1A, and CB1, with emerging therapeutic relevance in the management of osteoarthritis and chronic pain. **Conclusions**: Although clinical and preclinical research show promising results, clinical evidence is limited, and more studies should be performed in the future with isolated CBD.

## 1. Introduction

The hemp plant, also known as cannabis or botanically as *Cannabis sativa* L. (Cannabaceae), is a plant species with a historical use for thousands of years both for textile applications but also as a medicinal or food ingredient. It appears that its medicinal use dates to 2800 BC, when it was included in the *Chinese Emperor Shen Nung Pharmacopoeia*. In addition, the therapeutic properties of this species have been described in Hindu, Assyrian, Greek, and Roman texts, with clear indications for treating pain, inflammation, or lack of appetite.

Although cannabis is considered a monospecies (*Cannabis sativa* L.), there are different subspecies and varieties. The main varieties of cannabis are *Cannabis sativa f. chinensis* originating in China, *Cannabis sativa* var. *indica* originating in India, and *Cannabis sativa* var. *ruderalis* originating in Russia [1,2].

*Cannabis sativa* L. is an annual dioecious plant in which female and male flowers are found in separate organisms. It has a strong pivoting root (30/60 cm–2.5 m deep) and erect stems, which are usually angular, furrowed, and branched (opposite or alternative) with woody interior, varying from 1 to 6 m in height. The leaves are green and palmate (seven lobes) but the inflorescences consist of numerous flower heads that can be found on the long, leafy stems of each leaf armpit. The stamina (male flower) consists of five pale green hairy sepals about 2.5 to 4 mm long and five hanging stamens, with thin filaments and stamens. The pistillate (female flowers) are almost sessile and exist in pairs [3].

From a phytochemical point of view, the main compounds of pharmaceutical interest are cannabinoids, secondary metabolites with a unique structure that come from different biosynthetic pathways and therefore are usually referred to as terpenphenols due to the phenolic skeleton with isoprene units. In addition, we can find non-cannabinoid monoterpenes, sesquiterpenes, phenolic compounds of flavonoid type, and traces of alkaloids, fatty acids, and sterols. Terpenes are bioactive compounds whose main function is to favor the survival of the plant, the protection of predators, or the attraction to pollination, with 10 to 15 carbon atoms being the most abundant in Cannabis. The most abundant monoterpene is β-myrcene, which is an anti-inflammatory that acts by the E-2 prostaglandin pathway. The β-caryophyllene is the most common sesquiterpene in cannabis and it acts synergistically with some cannabinoids, producing antipruritic and anti-inflammatory effects. Among flavonoids, cannavaflavin A has also been positioned as an anti-inflammatory [4].

Within cannabinoids, tetrahydrocannabinol (THC) is the most common and characteristic chemical compound of the plant, together with CBD. It acts by receptor-dependent mechanisms CB1 (greater affinity than CB2) and CB2, which modulate pain, spasticity, sedation, appetite, and mood, in addition to being a bronchodilator, neuroprotector, antioxidant, and anti-inflammatory. It is considered the most important psychoactive component of the plant [4].

**Figure 1:**
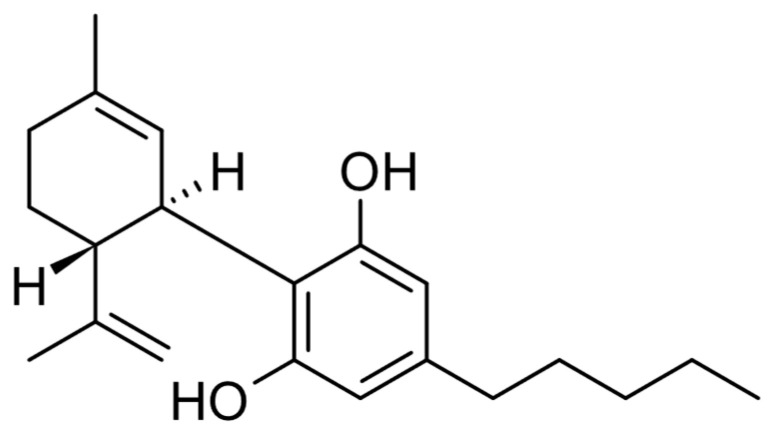
Chemical structure of CBD.

**Figure 2:**
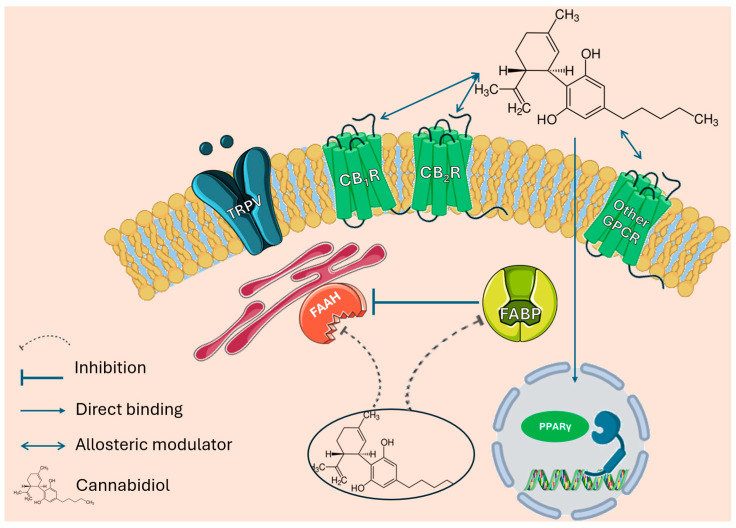
Mechanisms of action through which CBD exerts its effects at the pharmacological level.

Cannabidiol (CBD) (Figure 1) is the other cannabinoid that is found in the highest proportion with THC. CBD has a lower affinity for CB1 receptors compared to other cannabinoids like THC, and the literature indicates that CBD can still engage with CB1 receptors through allosteric modulation (Figure 2). CBD acts significantly through independent mechanisms with CB1 and CB2 receptors and has the unique ability to antagonize the CB1 receptor at low concentrations when THC is present [5,6,7,8,9]. CBD has a wide pharmacological activity from anticonvulsant, anti-inflammatory, and antioxidant to antipsychotic effects. In addition, it has the quality of decreasing some of the adverse effects of THC such as anxiety, hunger, tachycardias, and sedation. As for its mechanism of action, it seems to work through binding to different receptors coupled to protein G (modulation of CB1, CB2, 5HT1a, µ and δ opioid receptors), ionotropic receptors (TRPA1, TRPV1, TRPV2, TRPV4, 5HT3a, GABAA), enzymes (FAAH, LOX), nuclear factors (PPARγ), and transporters (FABP1, FABP3, FABP5, FABP7). CBD interacts with various GPCRs, specifically the µ-opioid receptor (MOR) and δ-opioid receptor (DOR). It exhibits binding affinities with inhibition constants (Ki) of 7000 nM for MOR and 10,000 nM for DOR. In contrast, CBD has a significantly higher affinity for dopamine D2 receptors, with a Ki of 11 nM, indicating functional high-affinity interactions with dopamine [4,10,11].

Naturally occurring cannabinoids allowed the discovery of the endocannabinoid system, consisting of endogenous cannabinoids, enzymes, and receptors; endocannabinoids are lipid mediators that bind to CB receptors. One of them is anandamide (AEA), which acts on the CB1, CB2, and TRPV1 receptors. It is mostly distributed in the brain and is part of sleep regulation, relaxation, feeding, neuroprotection, and immunomodulation. On the other hand, 2-arachidonoyl glycerol (2-AG) acts on presynaptic CB1 receptors to inhibit the release of neurotransmitters. AEA is degraded by fatty acid amide hydrolase (FAAH), and 2-AG by monoacylglycerol lipase (MAGL). The CB receptors are the CB1 receptor, found in the brain and the central nervous system, and the CB2 receptor, which is located in the cells of the immune system and in peripheral organs regulating other types of processes [4,10].

Besides the pharmacodynamic parameters, bioavailability is an important parameter of a drug’s understanding. As a part of pharmacokinetics, is essential to know the relation between the blood concentration and time. In this way, it is necessary to identify what route of administration is the most appropriate for CBD depending on the drug formulation. Oral administration has been the main route used in the majority of clinical trials, while the intraperitoneal route is the most indicated in preclinical trials for CBD administration [12,13,14,15]. Epidiolex^®^ is one of the CBD-containing drugs commercialized as an oral formulation. Several studies suggest that C_max_ and T_max_ depend on the dose [16,17]. However, other sources of oral CBD manifest a greater range in blood levels [18]. But bioavailability is not only essential to understanding the pharmacokinetics, but drug interactions are also important to comprehend, as medical CBD users are polypharmacy patients. Moreover, CBD is metabolized via the cytochrome P450 enzyme pathway, and it could modulate the immune system and increase the risk of viral infections and pneumonia and reduce blood pressure in patients treated with antihypertensives [19,20].

The main objective here in this work is to analyze preclinical and clinical evidence on CBD in monotherapy for pain treatment through a review following the PRISMA guidelines [21]. The secondary objectives are as follows:

- Analyze the safety of treatments in the clinical trials studied.
- Elucidate the mechanism by which CBD interacts to produce therapeutic effects through preclinical trials.
- Determine possible disease treatments based on their analgesic properties.

## 2. Results

**Figure 3:**
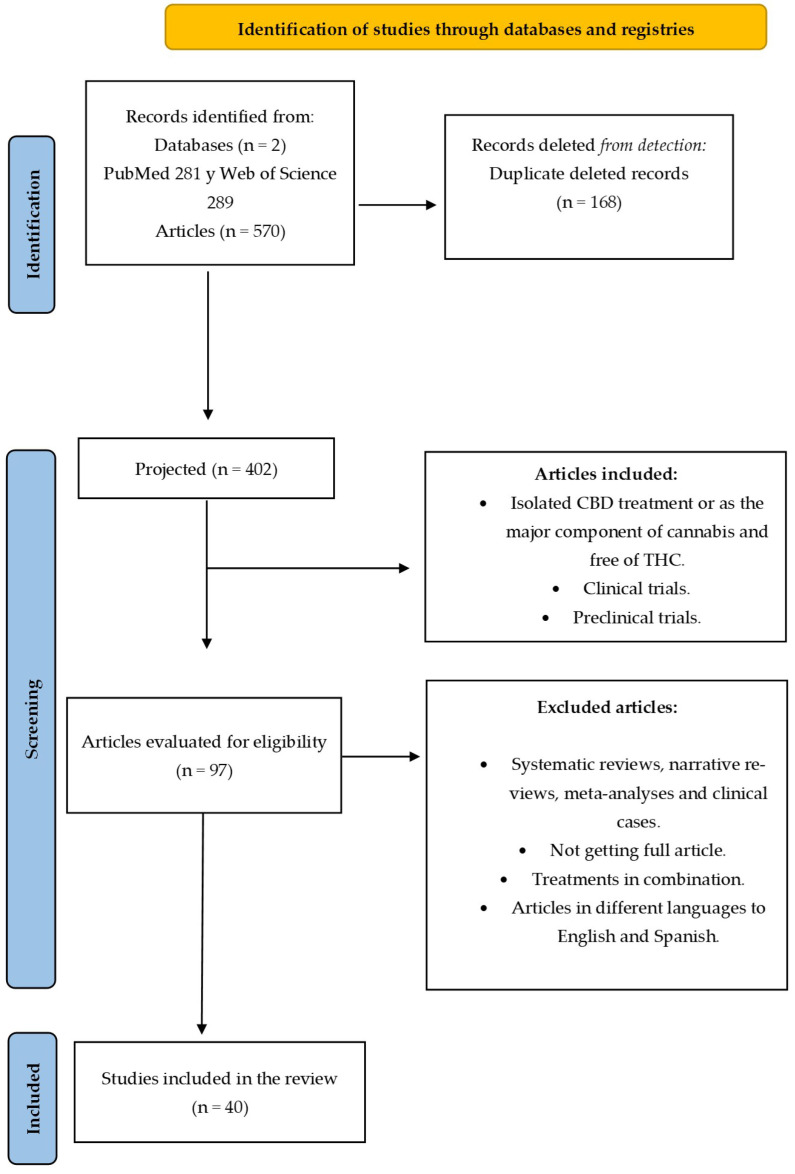
Flowchart depicting the study selection process following PRISMA guidelines.

Figure 3 shows the initial result after the search was 570 articles that a priori could be used for this systematic review, considering the two consulted databases. After a first screening and eliminating duplications, 402 articles were located. After deleting all the articles obtained with the search due to the noise of using the terms “(((cannabidiol) NOT (THC)) NOT (tetrahydrocannabinol)) AND (pain treatment)”, or those that did not use only CBD, such as studies with medicinal cannabis or drugs such as Sativex or nabiximol (extract with which Sativex is obtained) that are composed of CBD and THC, finally, a sample of 97 was selected from both databases; of these, 40 were used for the review due to the impossibility of finding the full article, and by deleting articles that seemed to fall within the criteria, but in which CBD was used in the treatment in combination therapy with other drugs.

The main reason for utilizing these criteria was to assess the presence of studies examining the efficacy of CBD in alleviating pain symptoms. Therefore, the importance of studies focusing on isolated CBD was vital, as they support the determination of whether CBD alone produces analgesic effects or if the therapeutic benefits stem from a combination of active ingredients. The final sample obtained was 40 articles, which were distributed as follows: 11 human clinical trials, 2 dog clinical trials [22,23,24,25,26,27,28,29,30,31,32,33,34], and 27 preclinical articles.

The final sample yielded three tables. Table 1 and Table 2 include details of the human and dog clinical trials, specifying the CBD treatment used, the number of participants, randomization status, and masking presence. These parameters were utilized to assign a Jadad score, aiding in evaluating the trial’s robustness and providing a comparative tool. Table 3 shows the treatments employed and the main conclusions of these preclinical trials.

## 3. Discussion

As previously mentioned, CBD can be administered intraperitoneally, intravenously, orally (oil, tablets), and topically (cream, oil, emulsion, gel). Depending on the type of administration (route and method), pharmacokinetic processes may be altered, meaning they can be faster or slower and may be magnified or not. There are five main components of pharmacokinetics: liberation, absorption, distribution, metabolism, and excretion (LADME). In veterinary and human practice, CBD is generally administered orally. A unique clinical trial performed in dogs estimated the oral bioavailability of CBD raw material capsules, finding it to be 19% lower compared to other forms without release. This low bioavailability is a crucial factor in determining and establishing an appropriate pharmaceutical dosage not only in animals but also in humans [61,62,63].

Inhalation is one of the fastest routes of administration for reaching plasma concentrations, thanks to the rapid gas exchange that occurs in the alveoli. Indeed, several studies concentrations confirm that CBD are reached promptly (3–10 min) and the inhaled bioavailability and the volumes of distribution (Vd) are higher than oral administration 31% and 10–15%, respectively [14,15,64,65,66]. In vitro studies with human skin have shown that the permeability of CBD is ten times higher than that of delta-9-THC and delta-8-THC, which explains the wide use of CBD-based products topically for pain treatment [67].

The metabolism of CBD is mainly carried out by different hepatic oxidase isoenzymes such as CYP2C19, CYP3A4, CYP1A1, and CYP2D6. Then, phase II conjugating pathways facilitate the elimination of the new metabolites by urinary excretion [13,66]. CBD exhibits a prolonged terminal elimination half-life, with the shortest observed following intravenous administration, followed by inhalation, while the longest elimination half-life occurs with oral administration [12,64]. American researchers concluded that selecting the appropriate CBD formulation is crucial, as pharmacokinetic profiles can vary significantly depending on whether it is administered sublingually, orally, or through smoking. This factor is considered even more critical than using pharmaceutical-grade CBD, provided the drug meets FDA standards for treating pain [68].

Out of the 11 clinical trials, 7 of them obtained results indicating that CBD treatment has pain-reducing properties [22,24,28,29,30,32,34] in osteoarthritis-related pain, chronic or neuropathic pain, bruxism, arthritis, and atopic dermatitis. In the latter four trials, the result was not achieved as expected, and the study conducted by Australian researchers did not exert significant differences respect to placebo [25]. Meanwhile, the administration of 150 mg of CBD oil every 24h in two doses did not deliver pain relief due to the performance of physical exercise in people unaware to training in Chicago (United States) [26]. A lower dose (50 mg of cannabidiol) was selected in another pharmaceutical form, such as chewing gum in a placebo-controlled intervention, but pain was not potentially reduced in patients with irritable bowel syndrome [31]. The article by Bebee et al. [25], despite having a high robustness and displaying no differences from placebo, cannot be used as evidence to rule out the analgesic/pain-reducing properties of CBD, as the study focuses on acute treatment for lower back pain in the emergency room, in which high-potency analgesics or opioids are used, so it is usual to observe no significant differences compared to a placebo. Thus, the absence of such variations does not necessarily imply a lack of analgesic properties. In addition, 83 patients with arthroscopic rotator cuff repair (ARCR) were treated with two different doses of CBD (25 and 50 mg) three times/day for 14 days. In this case, patients did not show significant deficits in pain, satisfaction or in patient-reported outcomes one year after surgery compared to a placebo group [33].

Otherwise, the two clinical trials conducted in dogs demonstrated great results in the treatment of osteoarthritis [23,27]. Both CBD oil doses (2 mg/kg) in capsules (20 mg/day) and unencapsulated (50 mg/day) showed positive results in dogs, even demonstrating anti-inflammatory effects [27] and improving the comfort of the animals. The latter was measured using the Canine Brief Pain Inventory (CBPI) and the Hudson Visual Analog Scale (HVAS) to assess the response to treatment compared to a placebo [23].

Nonetheless, there are five other clinical trials with great robustness that obtained results that support these analgesic properties [24,28,29,30,34]. According to the quality of the studies assessed by the Jadad scale, there are eight clinical trials with the maximum score of 5 points. Most of these works are relatively new and recent, since they have been carried out from 2020 to the present. Of all of them, up to five studies observe positive effects and one of them does not have superiority to placebo, and in many cases the topical route is an effective way for the application of CBD.

The work of Gamble [23] and that of Verrico [27] obtained scores of 4 and 5 points on the scale, respectively. With the first obtaining positive effects in pet animals treated with CBD orally and the second beneficial effects in treated humans with topical CBD. This scale allows us to observe that the majority of studies with a high Jadad score (≥4 points) conclude beneficial effects for CBD as an analgesic substance in certain pathological conditions related to pain. These properties are supported by all the results obtained in preclinical trials, and the pathways by which CBD acts in the body can be elucidated. It appears that the analgesic and anti-inflammatory properties are mediated through the activity on microglia [40,69], the decrease in pro-inflammatory cytokines associated with T cells, modulating their migration rate [53], the activation of TRPV-1 receptors [10,44,45,47,48,59], and the interaction with CB receptors. The decrease in neuropathic pain and part of its anxiolytic properties could be due to the activation of 5HT-1A receptors [10,36,44,45,49,60,70]. Due to their properties and mechanism of action, they could be a potential treatment for osteoarthritis, acute inflammatory eruptions, chronic pain and neuropathic pain, and other autoimmune diseases not only due to the properties mentioned above, but also due to its great safety, since no clinical or preclinical trials reported significant side effects nor treatment rejections. As for the pharmacokinetics, more studies would be needed to know which pharmaceutical form is more suitable for administration according to the pathology, either orally transported, or with no pharmaceutical form, or via the topical route. As such, they would make it clear that CBD is absorbed, distributed, and eliminated [23,27,46].

## 4. Materials and Methods

### 4.1. Search Strategy and Data Resources

To carry out this work, a systematic review was conducted following the PRISMA (Preferred Reporting Items for Systematic Reviews and Meta-Analyses) guidelines [21]. The data sources used in the review process were PubMed and Web of Science; the aim of the search was to find and analyze all the articles that were preclinical and clinical studies on CBD isolated or as a major component of cannabis without the presence of THC for the treatment of pain. The following terms were used for the search in both databases: “((cannabidiol) NOT (THC)) NOT (tetrahydrocannabinol)) AND (pain treatment)”. Terms such as CBD were not used, as it only caused noise in the search by adding articles dealing with common bile duct by sharing the same acronyms. In addition, in Web of Science we had to put an additional filter “(NOT (Sativex) NOT (nabiximol))”, eliminating articles dealing with Sativex and nabiximol, which despite being a CBD-based drug also contains THC. In PubMed, it was not necessary to apply the filter, since these terms were not included in the initial search, and adding those filters generated noise, resulting in a less specific search. This study was not registered in PROSPERO

### 4.2. Eligibility Criteria

The search was not limited by the year of publication, due to the limited number of preclinical and clinical studies performed only with CBD as the major component. So, all articles that assembled the inclusion and exclusion criteria were included until June of 2024. For this purpose, the following inclusion and exclusion criteria were used; the inclusion criteria were: clinical and preclinical trial articles (in vitro and in vivo) which were treated with CBD alone or as a major component of cannabis, in any pharmaceutical form and free of THC. The exclusion criteria included the presence of other cannabis components such as THC, systematic reviews, narrative reviews, meta-analyses, clinical cases, and articles not in English and/or Spanish. In addition to articles with impossibility of acquiring them in full text, the quality of the clinical trials was analyzed using the Jadad scale, which consists of an instrument to measure the risk of bias, particularly in pain research; each item of the scale is given a score of 1 or 0 points for each yes or no answer [71].

### 4.3. Selection Procedure

Two independent researchers (M.Y. and G.C.) performed the literature research and V.L. conducted supervision by including studies published until June 2024. Records were organized using Mendeley Reference Manager. Full texts were assessed using predetermined selection criteria

## 5. Conclusions

Based on the review carried out, there is clinical and preclinical evidence of CBD in the treatment of pain, so CBD is an effective and safe treatment in reducing pain due to its analgesic and anti-inflammatory properties. These effects seem to be mediated mainly by the activation of TRPV-1, 5HT-1A, and the allosteric modulation of CB1, showing great therapeutic promise in the management of osteoarthritis and chronic pain.

It would be interesting to increase the number of CBD studies, especially with a larger patient cohort. Many studies have been conducted using hemp extracts containing CBD, THC, and other cannabinoids, but it is particularly important and decisive to use products that are perfectly characterized in terms of the CBD proportion defining the absence or their psychoactive compounds. Based on the regulatory European market, CBD is orally approved as antiepileptic agent (Epidiolex^®^); nevertheless, there is room for improvement if new clinical trials are performed with CBD alone as an analgesic agent in different pain conditions.re

## Figures and Tables

**Table 1:** List of human clinical trials of CBD in pain management with Jadad score assignment.

Authors	Treatment	Number of Patients	Randomization	Masking	Jadad	Result
Cuñetti 2018 [22]	CBD 50–150 mg/2 day for 3 weeks.	7 patients with transplanted kidney.	No.	No.	1	Well-tolerated treatment; 6/7 patients had improvement in chronic pain; longer follow-up would be necessary.
Capano 2020 [24]	Oil rich in CBD, 60 mg daily.	131 patients with chronic pain.	Yes.	Double blind.	5	CBD is an effective analgesic that decreases opioid use in patients with chronic pain.
Xu 2019 [28]	Topical oil containing 250 mg of CBD.	29 patients with peripheral neuropathy sympathy.	Yes	Double blind	4	Topical application of CBD manages to significantly improve the pain and symptoms of peripheral neuropathy
Bebee 2021 [25]	400 mg CBD oral.	143 lower back pain patients.	Yes.	Double blind.	5	CBD was not superior to placebo as an adjunct medication to treat lower back pain.
Cochrane-Snyman 2021 [26]	150 mg CBD oil 24 and 48 h.	30 people who do not practice exercise.	No.	Double blind.	3	The results indicate that this dose and treatment time is not beneficial for muscle pain caused by exercise.
Gao 2022 [29]	JW-100 (pure CBD + aspartame) topical formulation for 14 days/twice a day.	18 patients with atopic dermatitis (AD).	Yes.	Double blind.	5	JW-100 showed statistically significant enhancements in AD symptoms after 14 days of topical application applied twice daily
Heineman 2022 [30]	1 mL of topical CBD (6.2 mg/mL) with shea butter.	18 participants with thumb basal joint arthritis.	Yes.	Double blind.	5	Topical treatment with CBD showed notable enhancements in pain and disability associated with thumb basal joint arthritis.
van Orten-Luiten 2022 [31]	50 mg CBD chewing gum.	32 females with irritable bowel syndrome (IBS).	Yes	Double blind.	5	Results show no significant differences in pain scores suggesting that perceived benefits did not outweigh practical obstacles such as prolonged chewing
Walczyńska-Dragon 2024 [34]	5 and 10% CBD formulations.	60 patients with temporo-mandibular disorders (TMD).	Yes.	Double blind.	5	Intraoral administration of CBD formulations demonstrated effectiveness in alleviating pain, reducing muscle tension, and decreasing bruxism activity in individuals with sleep bruxism and muscle-related TMDs.
Alaia 2024 [33]	25 mg and 50 mg of CBD 3 times/day for 14 days	83 patients with arthroscopic rotator cuff repair (ARCR).	Yes.	Double blind.	5	Perioperative CBD use for pain control in ARCR patients did not show significant deficits in pain, satisfaction, or patient-reported outcomes at one-year post-surgery compared to a placebo group.
Bawa 2024 [32]	Transdermal CBD gel (4% *w*/*w*) 3 times/day for four weeks.	15 patients with hand osteoarthritis.	No.	No.	1	Transdermal CBD gel potentially ameliorates pain and grip strength in individuals with symptomatic hand osteoarthritis (OA).

**Table 2:** List of animal clinical trials of CBD in pain management with Jadad score assignment.

Authors	Treatment	Number of Patients	Randomization	Masking	Jadad	Result
Gamble 2018 [23]	CBD oil 2 mg/kg.	14 companion dogs.	Yes.	Double blind.	4	Pharmacokinetic and clinical studies indicate that 2 mg/kg of CBD oil twice daily increases the comfort and activity of dogs with osteoarthritis.
Verrico 2020 [27]	Encapsulated CBD (20 mg day) and unencapsulated (50 mg day).	20 companion dogs with osteoarthritis.	Yes.	Double blind.	5	The results show that CBD has a high bioavailability and that it exerts anti-inflammatory properties in a solid and quantifiable way.

**Table 3:** Preclinical trials of CBD in pain models with experimental animals.

Authors	Model	Treatment	Main Result
Costa 2007 [35]	Male Wistar rats subjected to constriction of the sciatic nerve in right hind leg.	Oral CBD (20 mg/kg)	CBD reduced inflammatory mediators such as PGE2, iNOs, and lipid peroxide in several tissues.
Ward 2011 [36]	C57Bl/6 mice.	CBD (5.0 or 10.0 mg/kg IP)	CBD ameliorates allodynia and hyperalgesia in paclitaxel-induced neuropathic pain mice.
Hammel 2015 [37]	Male Sprague Dawley rats as a model of monoarthritic knee joint induced by Freund’s adjuvant.	Gel CBD (0.6-3.1-6.2-62.3 mg/day)	These data indicate that topical application of CBD has therapeutic potential for the relief of arthritis pain-related behaviors and inflammation without obvious side effects.
Giacoppo 2015 [38]	C57BL/6 mice experimental model of autoimmune encephalomyelitis (EAE).	1% of CBD-cream	Daily application of topical 1% CBD cream has the potential to provide neuroprotective benefits against the series of processes (inflammation, oxidative damage, and neuronal cell death) linked to the development of EAE.
Lehmann 2016 [39]	Non-obese diabetic female mice.	5 mg/kg CBD daily and five times weekly for ten weeks	CBD-treated mice with T1D exhibited delayed onset of the condition and demonstrated notably decreased inflammatory markers along with heightened functional capillary density (FCD) in the microcirculation of the pancreas.
Sajjadian 2017 [40]	Cuprizone-induced demyelination model in C57/ BL6 mice.	CBD injection 5 mg/kg	The results obtained that CBD attenuates the destructive effects of cuprizone on CC by decreasing oxidative stress and microglia. Microglia suppression will potentially reduce inflammatory lesions and limit demyelination within the CNS.
Genaro 2017 [41]	Wistar rats with an incision (pain model).	CBD intraperitoneal 3–10 mg/kg	There is evidence that CBD modulates the sensory and affective dimension of pain in a differential way.
Philpott 2017 [42]	Male Wistar rats with osteoarthritis (150–175 g) sodium monoiodoacetate model of osteoarthritis.	Local administration CBD 100–300 μg. Topical treatment with CBD	Local administration of CBD inhibits pain and peripheral sensitization in osteoarthritis. Topical CBD can be a safe treatment to treat pain in OA, as well as block acute inflammatory flare-ups.
Li 2018 [43]	Sham or contusion injury model in C57/BL6 mice.	Intraperitoneal CBD (1.5 mg/kg)	CBD significantly reduces - Pro-inflammatory cytokines and T cell-associated chemokines in mice with spinal cord injury. - The invasion of T cells into the damaged marrow.
De Gregorio 2019 [44]	Male Wistar rats (250–260 g) spared nerve injury model.	Oral CBD (10–40 mg/kg)	Low-dose CBD produces analgesia through the activation of TRPV1 receptors and reduces neuropathic pain through 5-HT.
Jesus 2019 [45]	Model of neuropathic pain induced by chronic constriction nerve injury in male Wistar rats.	Intraperitoneal CBD (0.1–0.3 mg/kg)	CBD can be a treatment for neuropathic pain in diabetics, acting through the activation of 5-HT1A.
Belardo 2019 [46]	Mild traumatic brain injury (TBI) induction in C57/BL6 mice.	10% CBD oil (30 μL)	Daily treatment with CBD significantly reduced pain, disappearing within 30 days.
Crivelaro do Nascimento 2020 [47]	Parkinson model by 6-OHDA in C57/BL6 mice.	Oral CBD (10, 20 and 100 mg)	CBD may be a useful drug to prevent parkinsonism-induced nociceptive pain by lowering its threshold. They also suggest that CB1 and TRPV1 receptors are important for CBD-induced analgesia. CBD could produce these analgesic effects by increasing endogenous anandamide levels.
Silva-cardoso 2021 [48]	Male Wistar rats (250 g) exposed to chronic constriction injury of the sciatic nerve.	CBD intraperitoneal 0.3, 3, 10, 30 mg/kg	The results may be clinically relevant for using CBD in the treatment of chronic pain and associated comorbidities due to interaction with CB1 and TRPV1 receptors.
Malvestio 2021 [49]	Albino Wistar rat model of neuropathic pain.	CBD injection (15, 30 and 60 nmol)	CBD could be a potential medication for pain and depression in patients with neuropathic pain. Due to its interaction with CB1 and 5-HT1A receptors.
Vivanco-Estela 2021 [50]	Parkinsonism-induced orofacial allodynia in Wistar rats.	CBD injection (10, 50 and 100 μg)	Local CBD treatment reduces the increase in allodynia and orofacial hyperalgesia in both sexes, although it responds differently to the same doses.
MLOST 2021 [10]	OA-induced male Wistar rats.	CBD injection (50 mg/kg)	The beneficial effect of CBD for osteoarthritis is mediated by the PPARy receptor, and the activation of the TRP channel by CBD is necessary to produce the analgesic effects.
Ding 2022 [51]	OA-induced adult mice (C57BL/6J).	CBD (1.0 mg/kg, i.v.)	The engagement of CBD with the serotonin 5-HT1A receptor plays a role in its pain-relieving and anxiety-reducing effects in the MIA-induced OA animal model.
Sepulveda 2022 [52]	C57BL/6 wild-type mice.	CBD (10 mg/kg, i.p.)	Treatment with CBD could potentially offer beneficial pain-relieving effects during the initial stage of chronic pain in formalin assay.
Li 2023 [53]	Sprague Dawley rats, model of pulpitis.	CBD (5 mg/kg i.p.)	The group treated with CBD did not show significantly higher sensitivity compared to the sham controls. Furthermore, CBD only attenuated a marker of macrophage activation (AIF-1).
Piao 2024 [54]	Sprague Dawley male rats with chronic prostatitis/chronic pelvic pain syndrome (CP/CPPS).	Oral CBD (50, 100 and 150 mg/kg)	CBD exhibits a notable ability to attenuate the pain associated with prostatitis, with a more pronounced enhancement observed at higher CBD dosage levels.
Marques 2024 [55]	Male Swiss mice.	CBD (20 mg/kg, i.p.)	Cannabidiol significantly reduced mechanical allodynia induced by paclitaxel.
Arantes 2024 [56]	Male and female Wistar rats.	Intraperitoneal CBD (0.3 and 3 mg/kg)	CBD demonstrates the capacity to mitigate enduring pain in individuals of both genders. In females, the responsiveness to CBD exhibits notable variations throughout the estrous cycle.
Rodrigues-Tavares 2024 [57]	Male Swiss mice.	Intraperitoneal CBD (0.3, 1, 10, or 30 mg/kg)	CBD administration attenuated pain hypersensitivity.
Jelínek 2024 [58]	Rheumatoid Arthritis rat model.	CBD-containing emulsion	Beneficial Impact of Cannabidiol (CBD) in a Rat Model of Rheumatoid Arthritis (RA).
Escobar-Espinar 2024 [59]	Male Wistar rats.	CBD (30 mg/kg)	CBD may provide therapeutic benefits for trigeminal neuralgia without inducing motor coordination deficits.
Zhu 2024 [60]	Male C57BL/6N mice.	CBD (30 mg/kg i.p.)	CBD exhibits antinociceptive effects by activating dopamine receptors and promoting wakefulness under sleep deprivation conditions.

## Data Availability

Data is contained within the article.
